# Supplementary material for: ER procollagen storage defect without coupled unfolded protein response drives precocious arthritis
Source: Life Sci Alliance. 2024 Jul 9;7(9):e202402842. doi: 10.26508/lsa.202402842 (PMC11234256; doi:10.26508/lsa.202402842)
Supplement: Supplementary file 1 [file LSA-2024-02842_TableS1.docx]

| Name | Sequence |
| --- | --- |
| Template oligodeoxynucleotide (ODN) sequence  (mutation in red) | 5'- CCAGCGACTCCCCAGCCTTCCCTGTGGTGACCACTCTTTCCTCACGACCTCTCTCTCTTGCAGGGTCCTCCTGGCCCCGTC**TCA**CCCTCTGGCAAAGATGGTGCTAATGGAATCCCTGGCCCCATTGGGCCTCCTGGTCCCCGTGGACGATCAGGCGAAACCGGCCCTGC-3' |
| COL2A1–1170 scrF | 5'-ACAAACATGAATCAGCCTCTCG-3' |
| COL2A1–1170 mutR  (only binds mutant sequence) | 5'-CATCTTTGCCAGAGGG**TGA**G-3' |
| COL2A1–1170 seqR | 5'-AGCCAGGATTGTGTGAAAGTGC-3' |
| COL2A1–1170 scrR | 5'-CAGCAGGAAACAGAGAGATCAGC-3' |
